# The Impact of Surgery on the Developmental Status of Late Preterm Infants – A Cohort Study

**Published:** 2015-01-10

**Authors:** Amit Trivedi, Karen Walker, Alison Loughran-Fowlds, Robert Halliday, Andrew J. A. Holland, Nadia Badawi

**Affiliations:** 1 Grace Centre for Newborn Care, The Children’s Hospital at Westmead, Sydney, NSW, Australia; 2 Sydney Medical School, The University of Sydney, Australia; 3 Douglas Cohen Department of Paediatric Surgery, The Children’s Hospital at Westmead, Sydney, Australia

**Keywords:** Late preterm infants, Neurodevelopmental outcome, Neonatal surgery

## Abstract

Aims: Despite increasing evidence in the literature regarding the impact of late prematurity on subsequent developmental impairment, the developmental outcome of late preterm infants who undergo major surgery remains unclear. The aim of this study therefore was to determine the developmental outcome for a cohort of late preterm surgical population.

Methods: Late preterm infants with a gestational age from 34-36 weeks inclusive who were enrolled in the state-wide prospective Development After Infant Surgery (DAISy) study and who had undergone non-cardiac major surgery within the first ninety days of life were eligible for inclusion. Infants were assessed at one and three years of ages.

Results: Forty-six infants were enrolled in the study, of which 38 infants had a complete developmental assessment at one year of age. Of these infants, late preterm infants scored significantly lower than the standardized norms of the assessment on the expressive language and gross motor subscales. At three years of age 26 infants were reassessed: late preterm infants who underwent major surgery only scored significantly lower than the standardized norms on the cognitive subscale (p less than 0.001).

Conclusions: These data provide the evidence that late preterm infants who undergo major non-cardiac surgery are at risk of developmental impairment and consideration should be given to enrolling this cohort in multi-disciplinary developmental follow-up clinics.

## INTRODUCTION

It is well recognised that extremely preterm infants, defined as less than 28 weeks gestation, are at risk of developmental impairment [1], which justifies their eligibility in neonatal developmental follow-up programs. There is now increasing evidence in the literature that late preterm infants of 34 to 36 weeks of gestation are also at a higher risk of developmental delay, including cerebral palsy, than term infants. [2-5] This may result in long term developmental sequelae.[4,6] Moreover, the infants who undergo major surgery within the first few months of life are at risk of developmental delay.[7,8] It would seem logical that late preterm infants would be at equal or greater risk when undergoing major surgical procedures in early infancy. 

Despite increased awareness of late preterm infants and the outcomes of surgery, there remains a paucity of data documenting the developmental outcomes of preterm infants who have undergone major surgery. Thus we aimed to investigate this question and describe the developmental outcome for a cohort of late preterm surgical infants.

## MATERIALS AND METHODS

Late preterm infants with a gestational age from 34-36 weeks inclusive who were enrolled in the state-wide prospective Development After Infant Surgery (DAISy) study and who had undergone non-cardiac major surgery within the first ninety days of life were eligible for inclusion. Non-cardiac major surgery was defined as opening of a body cavity. Infants with a known chromosomal anomaly, which caused developmental impairment, were excluded, as were those who required neurosurgery. Surgical infants enrolled in this study were recruited from the three Children’s Hospitals in New South Wales (NSW). No other surgery to our knowledge is performed at other hospitals in New South Wales.

At one year of age (corrected for prematurity) and then again at three years of age, the children were assessed using the Bayley Scales of Infant and Toddler Development, Version-III (Bayley III). This assessment is currently the developmental assessment of choice, used in all neonatal follow-up clinics in NSW. It consists of five scales: cognition, receptive and expressive language, and gross and fine motor. This standardized test of child development is age-normed to have a mean of 10 and a standard deviation (SD) of 3 for each subset. Mild developmental delay was defined as a score between 1 to less than 2 SD below the mean; moderate delay, as 2 to less than 3 SD below the mean; and severe delay, as 3 SD below the mean. The children were assessed on each of the subscales and the mean scores compared with the standardized norms of the assessment.

Ethics approval was obtained from each recruitment site and informed consent obtained from the parents or guardian. Statistical analyses were performed using SPSS version 21 and consisted of comparing the mean scores between the groups using one sample t-tests with p less than 0.05 considered statistically significant.

## RESULTS

Forty-six infants with a gestational age from 34-36 weeks inclusive were enrolled in the surgery group and were eligible for inclusion in this study. Five infants were lost to follow-up, one died prior to assessment, one was withdrawn and one infant with Trisomy 21 was excluded, leaving 38 infants who had complete developmental assessments. More infants were female (22 vs 16) with a mean maternal age of 27.3 years (range 15-51 years). The majority of infants required surgery for gastrointestinal conditions such as gastroschisis and atresias (Table 1). Half of the infants were born by Caesarean section, four had instrumental deliveries and the remaining 15 were born by a normal vaginal delivery. 

**Figure:**
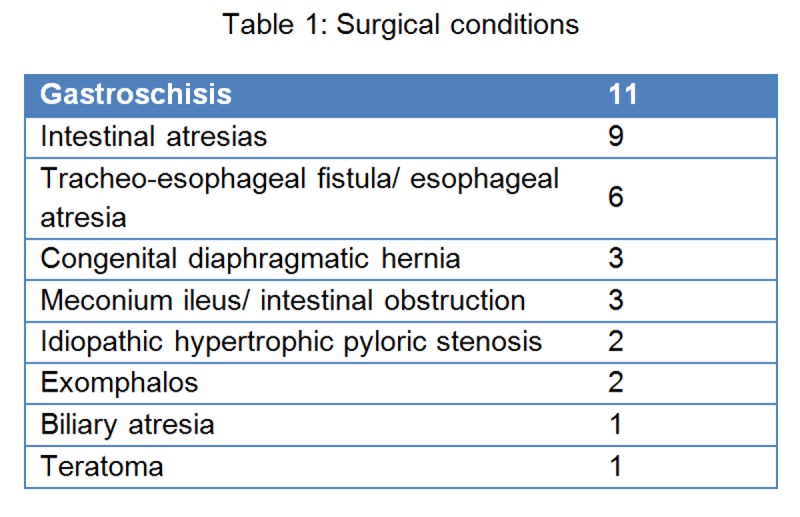
Table 1: Surgical conditions

Late preterm infants who had major surgery scored significantly lower than the standardized norms of the assessment on two of the subscales at one year of age: expressive language and gross motor (p less than 0.05, Table 2).

**Figure:**
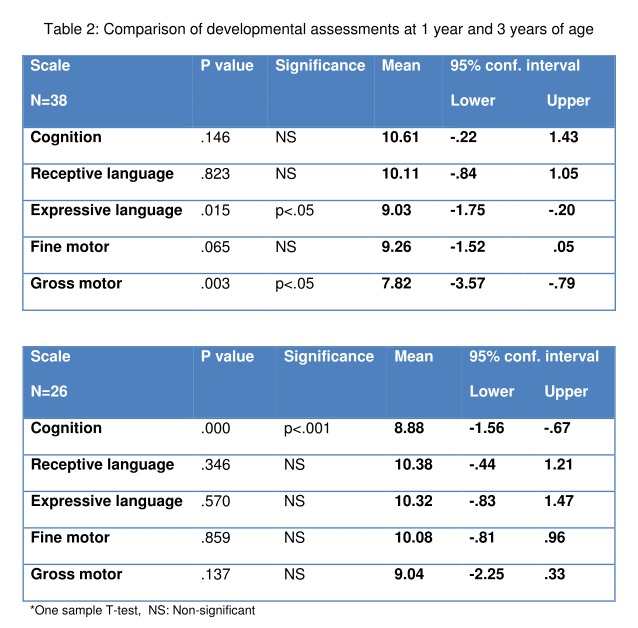
Table 2: Comparison of developmental assessments at 1 year and 3 years of age

Of these 38 infants assessed at one year of age, 10 children were subsequently lost to follow-up, one was withdrawn and one child died, leaving 26 infants who returned for a three-year assessment. There was no difference between those infants who returned for assessment and those who did not, in terms of socio-demographic and clinical factors. At three years of age, the late preterm infants who underwent major surgery only scored significantly lower than the standardized norms for the cognitive subset (p less than 0.001, Table 2).

This finding at three years was unexpected, so we then reviewed the one year outcomes for the 26 infants who were re-assessed at three years of age and found that, at one year of age, these children were only significantly lower than the normative mean in the gross motor subtest. (p less than 0.05, Table 3).

**Figure:**
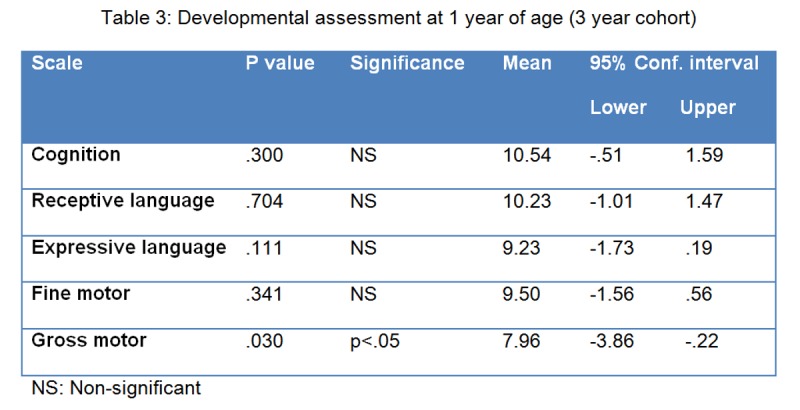
Table 3: Developmental assessment at 1 year of age (3 year cohort)

## DISCUSSION

This is the first prospective study that we have found in the English language literature that focuses on the late preterm infant group who have undergone major non-cardiac surgery. We have shown that late preterm infants who undergo non-cardiac surgery in the newborn period are at increased risk of developmental impairment at one year of age especially in language and motor skills. Some of these problems persist or change over time: cognition remained significantly delayed at three years of age whereas expressive language and gross motor skills improved. 

The delay in language at one year of age is concerning. A paper by DeMauro et al has expressed concern that late preterm infants have more oro-motor dysfunction at one year of age.[9] Infants who undergo major surgery, who may experience prolonged periods of total parenteral nutrition and the inability to feed orally, are also known to have poor oro-motor function. This may be associated with oral aversion and an increased risk of language delay at one year in term infants.[7] Therefore, the combination of being preterm and undergoing major surgery might be expected to impact even more on future language development. Among the 26 infants who were followed to three years, language scores were within the average ranges, although the Bayley III does not assess difficulties with pronunciation and articulation that become more obvious at this age.

Cognitive delay at three years of age was concerning as poor cognitive skills can be problematic for later school entry. Late preterm infants who have not undergone surgery already are at risk of worse educational outcomes and need for learning support at school.[10-13] Gross motor skills were delayed at one year which is consistent with findings in term infants who require surgery.[7] Interestingly, gross motor skills were not delayed at age three. This may be because infants who had gross motor difficulties at age one are usually enrolled in community or outpatient physiotherapy programmes which may improve their skills before being reassessed, however only three infants had been enrolled in physiotherapy. 

Surgery and anaesthesia may play a part in increasing an infant’s risk of poor developmental outcomes, although it is likely that the aetiological pathophysiology has multiple contributing factors. There has been increasing interest in the possible neurotoxic effects of anaesthetics on the developing brain, however the evidence remains disputed.[14,15] Certain studies have shown that term infants are at increased risk of neurodevelopmental problems following non-cardiac major surgery.[7] Therefore, the combination of both prematurity and need for surgery would seem likely to place infants even more at future risk of neurodevelopmental impairment. It is unclear whether these outcomes are related to surgery, the underlying congenital anomaly or late preterm birth, thus further research is required. 

In addition, mothers of late preterm infants are more likely to undergo Caesarean sections and have other antenatal complications,[2,16] with increased risk of medical complications and the need for ventilator support.[2,4,16,17] These insults impact on the late preterm infant during a period of critical brain development and growth, with increased risk of poor developmental outcomes.[10,18] As a significant proportion of infants (56.7%) are delivered for non-evidence-based reasons, reasons for late preterm birth thus need to be re-evaluated and balanced against risks of mortality with delayed births at term.[19,20]

It is clear from our results that late preterm infants would benefit from multidisciplinary follow-up to identify early any developmental problems, so they can be linked into appropriate community services. We and others have previously recommended that term infants undergoing surgery should be enrolled in developmental follow up programmes.[7,21-23] At risk infants streamlined into early intervention programmes have been shown to have improved cognitive development by up to 25% in addition to reducing adverse social outcomes such as school absenteeism, delinquency, and long-term unemployment.[24] Thus, it is vitally important that late preterm infants are also given this opportunity to improve their long term outcomes. 

Limitations: As we and others have reported previously, the Bayley Scales of Infant and Toddler Development (version-III) may underestimate developmental delay in the Australian population, therefore the results for this cohort may actually be worse than the scores reflect.[25-27] The Bayley III, like other developmental tests, may also have limited sensitivity at one year of age. Another limitation is the fact that we did not have a non-surgical late preterm group for comparison. As is found in many longitudinal studies we had a considerable loss to follow-up (reference attrition), however there was no difference between the groups in demographics and clinical factors.

## Conclusion

In conclusion, late preterm infants who require major surgery in infancy are a potentially neglected group of intensive care admissions. These data provide the evidence that they are at risk of long-term developmental impairment and consideration should be given to enrolling this cohort in multi-disciplinary developmental follow-up clinics. The decision to deliver an infant early should be made cautiously examining carefully the need for preterm birth and bringing evidence based medicine into the forefront. As these are early developmental outcomes, the problems reported may reflect developmental delay rather than long-term impairment, thus follow-up into childhood is essential.

## Footnotes

**Source of Support:** Funded by March of Dimes Birth Defects Foundation, Project Grant #12-FY06-232

**Conflict of Interest:** None

